# Exome sequencing identifies *HELB* as a novel susceptibility gene for non-mucinous, non-high-grade-serous epithelial ovarian cancer

**DOI:** 10.1038/s41431-025-01786-0

**Published:** 2025-02-12

**Authors:** Ed M. Dicks, Jonthan P. Tyrer, Suzana Ezquina, Michelle Jones, John Baierl, Pei-Chen Peng, Michael Diaz, Ellen Goode, Stacey J. Winham, Thilo Dörk, Toon Van Gorp, Anna De Fazio, David D. L. Bowtell, Dale W. Garsed, Kunle Odunsi, Kirsten Moysich, Marina Pavanello, Florentia Fostira, Penelope M. Webb, Jana Soukupová, Paul A. Cohen, Weiva Sieh, Renée Turzanski Fortner, Charite Ricker, Beth Karlan, Ian Campbell, James D. Brenton, Susan J. Ramus, Simon A. Gayther, Paul D. P. Pharoah

**Affiliations:** 1 Department of Public Health and Primary Care, University of Cambridge, Cambridge, UK; 2 Department of Biomedical Sciences, Cedars-Sinai Medical Centre, Los Angeles, CA USA; 3 Department of Computational Biomedicine, Cedars-Sinai Medical Centre, Los Angeles, CA USA; 4 Mayo Clinic, Rochester, MN USA; 5 Gynaecology Research Unit, Hannover Medical School, Hannover, Germany; 6 Division of Gynaecological Oncology, Leuven Cancer Institute, University Hospital Leuven and KU Leuven, Leuven, Belgium; 7 Centre for Cancer Research, The Westmead Institute for Medical Research, Sydney, NSW Australia; 8 The Daffodil Centre, The University of Sydney, A JOINT Venture with Cancer Council NSW, Sydney, NSW Australia; 9 Department of Gynaecological Oncology, Westmead Hospital, Sydney, NSW Australia; 10 Peter MacCallum Cancer Centre, Melbourne, VIC Australia; 11 Sir Peter MacCallum Department of Oncology, The University of Melbourne, Parkville, VIC Australia; 12 University of Chicago Medicine Comprehensive Cancer Center, Chicago, IL USA; 13 Division of Cancer Prevention and Control, Roswell Park Comprehensive Cancer Center, Buffalo, NY USA; 14 School of Clinical Medicine, Faculty of Medicine and Health, University of NSW, Sydney, NSW Australia; 15 Human Molecular Genetics Laboratory, National Centre for Scientific Research, Athens, Greece; 16 QIMR Berghofer Medical Research Institute, Brisbane, QLD Australia; 17 Institute of Medical Biochemistry and Laboratory Diagnostics, First Faculty of Medicine, Charles University and General University Hospital in Prague, Prague, Czechia; 18 Division of Obstetrics and Gynaecology, Medical School, University of Western Australia, Crawley, WA Australia; 19 MD Anderson Cancer Center, Houston, TX USA; 20 Division of Cancer Epidemiology, German Cancer Research Center, Heidelberg, Germany; 21 Department of Research, Cancer Registry of Norway, Norwegian Institute of Public Health, Oslo, Norway; 22 Keck School of Medicine, Division of Medical Oncology, University of Southern California, Los Angeles, CA USA; 23 University of California Los Angeles, Los Angeles, CA USA; 24 Department of Oncology, University of Cambridge, Cambridge, UK; 25 Adult Cancer Program, Lowy Cancer Research Centre, University of NSW, Sydney, NSW Australia; 26 Center for Inherited Oncogenesis, Department of Medicine, UT Health San Antonio, San Antonio, Texas USA

**Keywords:** Risk factors, Genetic association study

## Abstract

Rare, germline loss-of-function variants in a handful of DNA repair genes are associated with epithelial ovarian cancer. The aim of this study was to evaluate the role of rare, coding, loss-of-function variants across the genome in epithelial ovarian cancer. We carried out a gene-by-gene burden test with various histotypes using data from 2573 non-mucinous cases and 13,923 controls. Twelve genes were associated at a False Discovery Rate of less than 0.1 of which seven were the known ovarian cancer susceptibility genes *BRCA1*, *BRCA2*, *BRIP1*, *RAD51C*, *RAD51D, MSH6* and *PALB2*. The other five genes were *OR2T35, HELB, MYO1A* and *GABRP* which were associated with non-high-grade serous ovarian cancer and *MIGA1* which was associated with high-grade serous ovarian cancer. Further support for the association of *HELB* association comes from the observation that loss-of-function variants in *HELB* are associated with age at natural menopause and Mendelian randomisation analysis shows an association between genetically predicted age at natural menopause and endometrioid ovarian cancer, but not high-grade serous ovarian cancer.

## Introduction

Substantial progress has been made in the past 30 years in identifying inherited genetic variation associated with an increased risk of epithelial ovarian cancer (EOC). The “high-penetrance” genes *BRCA1* and *BRCA2* were identified by linkage studies in the 1990’s; protein truncating variants in these genes confer a substantial lifetime risk of epithelial ovarian cancer as well as breast cancer and other cancers. Epithelial ovarian cancer is also known to be part of the Lynch Syndrome phenotype associated with protein-truncating variants in the mis-match repair genes. Rare coding variants in *BRIP1, PALB2, RAD51C* and *RAD51D* have been shown to confer more moderate risks by using candidate-gene case-control sequencing [1–3]. Also, over the past 15 years, large-scale genome-wide association studies (GWAS) conducted by the Ovarian Cancer Association Consortium (OCAC) have identified more than 40 common susceptibility alleles [4, 5].

There are five major histotypes of epithelial ovarian cancer—high-grade serous, low-grade serous, clear cell, endometrioid and mucinous—which share some of the heritable component of disease risk [6]. Nevertheless, there are some notable differences in their genetic risk factors. High- and moderate-penetrance risk variants in *BRCA1*, *BRCA2*, *BRIP1*, *RAD51C* and *RAD51D* predispose to high-grade serous EOC whereas loss-of-function variants in the mis-match repair genes predispose to endometrioid and clear cell EOC. There are also histotype-specific differences in the risks conferred by common risk alleles with the mucinous histotype in particular being substantially different from the other histotypes [4, 5].

The uncommon and rare, high- and moderate penetrance alleles identified to date explain about one-quarter of the inherited component of epithelial ovarian cancer susceptibility with a further 5% explained by the known common risk alleles. Genome-wide heritability analyses have estimated that the set of common variants that are tagged or captured by the standard genome-wide genotyping arrays explains about 40 percent of the familial aggregation –the so-called chip heritability. The characteristics of the alleles that account for the remaining familial aggregation are not known; analyses of whole-genome data suggest that a substantial portion is explained by rare variants. Only a small fraction of genes, mostly those involved in DNA repair, have been examined for risk association using the large sample sizes needed to detect modest risks. Hence, there could be many more genes conferring similar risks yet to be discovered. The aim of this project was to identify genes with rare coding variants that confer loss of function (LoF) that are associated with risk of epithelial ovarian cancer.

## Methods

### Description of case and control datasets

Germline whole exome sequencing (WES) data and whole genome sequencing (WGS) data as BAM or CRAM files from multiple epithelial ovarian cancer case series were collated from multiple sources (Table 1). Control sequencing data were sourced wholly from the UK Interval study; a set of healthy UK blood donors (https://www.intervalstudy.org.uk/). All analyses restricted case histotypes to high-grade serous, low-grade serous, clear-cell, endometrioid, mixed, and other rare histotypes. Mucinous ovarian cancer cases were excluded because it has previously been shown that the genetic aetiology of this histotype differs substantially from the other histotypes [5]. In total, exome or whole genome sequencing data were available for 1638 cases and 4502 controls. We also used the variant calls (as VCF files) for 1099 EOC cases and 9423 cancer free controls from the WES sequencing released by UK Biobank (UKB) (https://www.ukbiobank.ac.uk/). Cases were individuals with a diagnosis of invasive epithelial ovarian cancer (ICD10 code C56) with clear cell, endometrioid, papillary, other and serous histology codes. Controls were age matched women without a cancer diagnosis and without a history of oophorectomy. Up to ten controls were selected for each case. Thus, the final sample size was 2737 cases and 13,925 controls before sequencing quality control.

**Table 1:** Number of non-Biobank epithelial ovarian cancer patients by source of sequencing (total before sample QC 1638; total after QC 1474).

Data source	Case type	Number (before/after QC)	Reference	Accession number
*Exomes*				
UK Familial Ovarian Cancer Registry	BRCA1/2-negative with family history	53/42	Unpublished	
Leuven	Unselected	45/44	Unpublished	
Gilda-Radner Ovarian Cancer Registry	BRCA1/2-negative with family history	96/80	Unpublished	
OCAC	Positive family history or <50 years of age	262/232	Unpublished	
Mayo Clinic	Unselected	25/24	Unpublished	
Campbell	BRCA1/2-negative high-grade serous	536/493	[40]	EGAD00001006030
Hannover	Unselected	11/7	Unpublished	
TCGA	High grade serous	413/361		phs000441.v2.p6
*Whole genomes*				
Peter MacCallum Cancer Centre	High-grade serous	93/92	[41]	EGAD00001000877
Bowtell	Long-term survivors with high-grade serous	48/46	[42]	EGAD00001009398
BRITROC	High grade serous	56/53	[43]	EGAD00001004189

### Variant calling and filtering

All BAM/CRAM files were aligned to human genome version hg19/GRCh37. The original TCGA EOC BAM files had been aligned against human genome hg18/NCBI36, these data were lifted over to build hg19 with the CrossMap s/w [7] to match the rest of the WES/WGS data. All non-Biobank sequencing data were analysed in an identical way. Duplicate sequence reads were removed with the *picard* sequencing tools [8]. Sequence reads were partitioned per chromosome and general manipulation performed with the samtools s/w [9]. Variants were called with the Genome Analysis ToolKit (GATK) UnifiedGenotyper version 3.8-1 [10], and following the best practices guide as appropriate to our data [11, 12]. We restricted our risk variant discovery to substitutions and short indels (length <= 12 bp). Variants were annotated with *ANNOVAR* [13] referred to the UCSC RefSeq gene transcript set (https://genome.ucsc.edu). Protein coding transcripts with an NM_* type identifier were used, with the transcript having the longest coding sequence being chosen for genes with multiple transcripts. This yielded 19,092 gene transcripts at human reference hg19/GRCh37 for variant annotation. The averaged coverage of targeted bases at 10X for the non-UKB samples was 91 percent for cases and 92 percent for controls.

UK Biobank VCF calls were based on build GRCh38 [14]; in order to incorporate these data into our pipeline we lifted over these calls to build GRCh37 using *picard* and inserted the data at the appropriate step. Only Biobank VCF calls with depth (DP) greater than or equal to 10 and genotyping quality (GQ) greater than or equal to 20 were retained.

Variant calls from GATK were filtered with an in-house hard filter tuned for optimum sensitivity by comparison of WES calls with chip genotyping calls from multiple genotyping arrays [see Chip Genotyping Data for details]. Additionally for all call sets, variants were carried forward only if depth (DP) >= 10 and alternate allele frequency (AAF) >= 15 percent. A more stringent filter was also applied, assigning calls a high quality (HQ) having AAF > = 20 percent and number of alternate alleles >= 4. All variant sites with at least 1 occurrence of an HQ call were retained, whilst sites without any HQ calls were rejected.

A rare variant was defined as one with minor allele frequency (MAF) <= 0.1 percent in non-UKB controls and cases combined. Each variant was visually inspected with the Integrative Genomics Viewer (IGV) software [15] and rejected if any doubts raised, e.g. not called bidirectionally. Visual inspection of variants called for UK Biobank was only carried out for those variant sites not validated in the non-Biobank data.

QC was applied to each rare variant site, rejecting sites with genotype frequencies showing significant deviation from those expected under Hardy-Weinberg equilibrium in either cases or controls (*p*-value < 10^−15^ and for UKB and non-UKB separately), and those with missingness >20% (proportion of samples with depth<10). We also tested each variant for association with epithelial ovarian cancer and excluded those with test for association *p*-value <= 10^−7^ and 0 rare control alleles; the threshold was chosen to exclude variants with effect sizes greater than those for *BRCA1* pathogenic variants as these are unlikely to be true positives.

### Variant classification

Variants were defined as loss-of-function according to the following criteria: 1) Variants predicted to cause protein truncation, that is stopgain variants, frameshift indels, and canonical splice site variants. 2) Non-canonical splice site variants and in-exon variation within 3 bp of the exon-splice boundary predicted by the MaxEntScan algorithm to disrupt splicing [16]. Qualifying variants with a wild-type MaxEntScan score greater than or equal to 3 and decreased by greater than 40 percent in comparison to the reference sequence were assumed to be deleterious. 3) Missense single nucleotide variants or in frame indels designated by multiple submitters to the NCBI ClinVar database (https://www.ncbi.nlm.nih.gov/clinvar) as either pathogenic or likely pathogenic with no conflicts between submitters.

### Sample quality control and exclusion

Samples were removed if they met any of the following criteria: i) low average depth of coverage (<25% at 10×) ii) excess LoF calls (>1000) iii) concordance of exome data variant calls and chip genotyping calls (see below) of <95% iv) known duplicates or cryptic duplicate sample based on common variant calls. After exclusions, a total of 1474 cases and 4500 controls remained in the non-Biobank set and 1099 cases and 9423 controls in the Biobank set (Table 1 and Table 2).

**Table 2:** Number of epithelial ovarian cancer patients by histotype after QC.

Histotype	Non-UKB	UKB	Total	% of total
High-grade serous^a^	1237	869	2106	81.8
Low-grade serous	52	0	52	2.1
Clear cell	52	86	138	5.4
Endometrioid	110	139	249	9.7
Mixed/unknown	23	5	28	1.1

^a^Includes those with carcinosarcoma and serous carcinoma of unspecified grade.

### Chip genotyping data

We used chip genotype calls to tune filters for rare variant calling and check integrity of sample naming and also as an additional level of QC for any non-UKB samples overlapping chip manifests. Data were from four different Ovarian Cancer Association Consortium genotyping projects (OncoArray [4], iCOGS [17], exome chip [18], and an ovarian GWAS [19]), and TCGA. The numbers of WES/WGS samples overlapping with each chip were 323, 350, 81, 95, and 412, respectively.

### Statistical methods

We carried out a gene-by-gene simple burden test for the association of rare loss-of-function variants with all non-mucinous ovarian cancer, high-grade serous ovarian cancer, and non-high-grade serous ovarian cancer. Rare variants were defined as those with a minor allele frequency of less than 0.1% in the non-UKB dataset. We classified each individual for each gene as a loss-of-function variant carrier or non-carrier, depending on whether they had at least one rare variant (below the MAF threshold) in that gene or not. Then we performed a logistic regression for each gene adjusting for the top four principal components to account for cryptic population structure and genetic ancestry. Principal component analysis for the non-UK Biobank data was carried out using data from 36,047 uncorrelated variants (pairwise *r*^2^ < 0.1) with MAF > 0.03 using an in-house programme (available at http://ccge.medschl.cam.ac.uk/software/pccalc/). Principal components for the UK Biobank samples were provided by UK Biobank [20]. We also adjusted for study stratum—non-UKB, UKB 50 K sample set and UKB non-50K sample set. The UK Biobank data were stratified on recommendation from UK Biobank, since different oligo lots had been used in the 2 stages of UK Biobank sample sequencing.

We calculated a false discovery probability based on the methods of Benjamini and Hochberg [21] and a Bayes False Discovery Probability using the method proposed by Wakefield [22]. For the latter method, we assumed a prior probability of association for any one gene of 0.005 – ~100 expected true number of genes truly associated with epithelial ovarian cancer—and a likely maximum effect size (log odds ratio) of 0.836.

We applied Mendelian randomisation using five different methods implemented in the R package *TwoSampleMR* (MR Egger, weighted median, inverse variance weighted, simple mode and weighted mode) [23]. Five methods were used because each method is susceptible to different possible biases and consistent finding using different methods provide stronger evidence for any observed association. Genetic instrumental variables were derived from summary statistics for association between common genetic variants and epithelial ovarian cancer by histotypes published by the Ovarian Cancer Association Consortium [5] (available at www.ebi.ac.uk/gwas/publications/38723632). Power for the Mendelian randomisation analyses was calculated using the method reported by Brion [24]. Other analyses were conducted using the *patchwork* [25] and *tidyverse* [26] packages of the *R* software [27] implemented in *R Studio* [28].

## Results

**Fig. 1:**
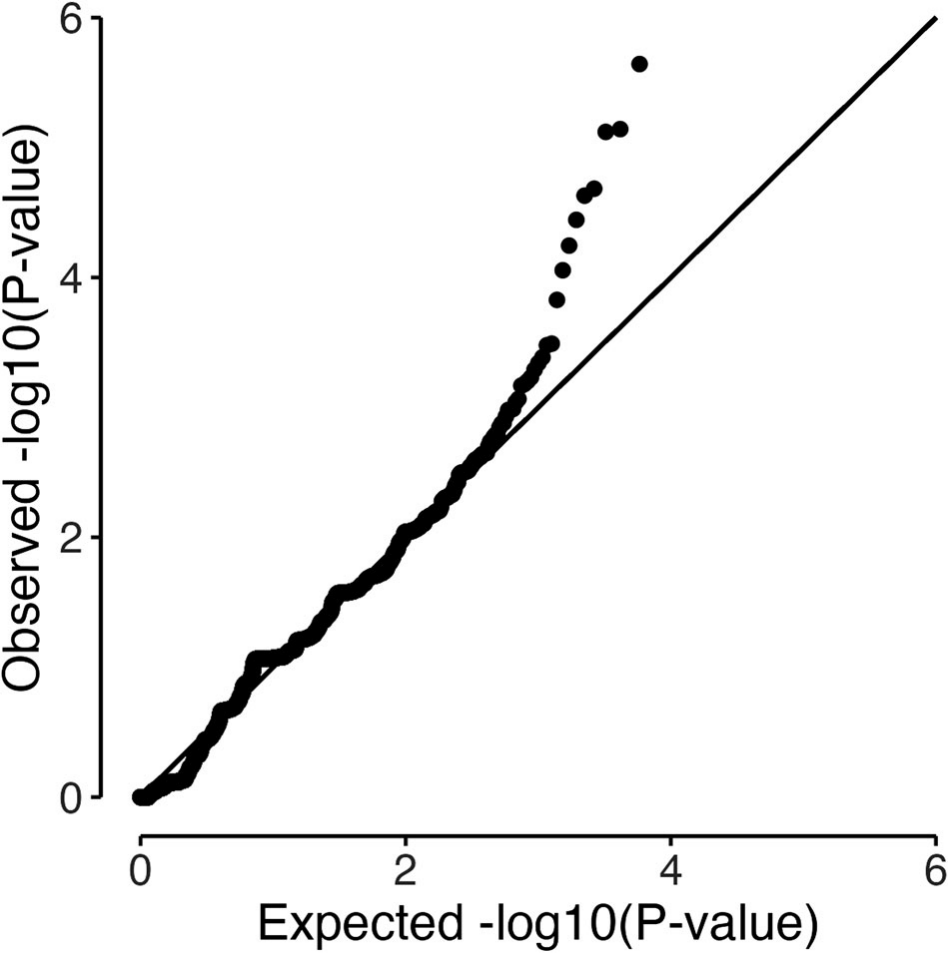
QQ plot for association analysis of genes with at least one case or control loss-of-function variant carrier in the non-UK Biobank data set (three genes with smallest *P*-values excluded).

An initial analysis was performed for all genes in the non-UKB set of 1474 cases and 4500 controls that passed QC. There were 12,761 genes with at least one case or control loss-of-function variant carrier with minor allele frequency of less than 0.1 percent, of which 4623 had sufficient pathogenic variant carriers to obtain a risk estimate. There was little evidence of inflation of the test statistic (Fig. 1) showing that the potential bias arising from systematic differences in sequencing between cases and controls has been prevented by the data harmonisation process. Seven hundred and thirty-seven genes had a *P*-value for association of less than 0.05; these genes were selected for additional analysis in the UKB data in addition to candidate genes *ATM*, *BARD1*, *CHEK2*, *FANCM*, *MLH1*, *MSH2*, *MSH6*, *PMS2*, *RAD51B*, *SLX4*, *TIPARP* and *TP53* which have previously been confirmed or suggested as ovarian cancer susceptibility genes [1–3, 29].

The results of the simple burden test for association of rare loss-of-function variants in each gene with non-mucinous ovarian cancer, high-grade serous ovarian cancer and non-high-grade serous ovarian cancer using the combined data are shown in Supplementary Table 1 with the results for non-UKB and UKB shown in Supplementary Table 2. The association from the combined data with the smallest P-value from the three histotype-specific analyses was selected for each gene and the genes were then ranked by *P*-value. Table 3 shows the 31 genes associated with ovarian cancer at a False Discovery Rate of less than 0.2. Twelve genes were associated at a False Discovery Rate of less than 0.1, of which seven were the known ovarian cancer susceptibility genes *BRCA1*, *BRCA2*, *BRIP1*, *RAD51C*, *RAD51D, MSH6* and *PALB2*. The other five genes were *OR2T35, HELB, MYO1A, GABRP* and *MIGA1*. *BRCA1*, *BRCA2*, *BRIP1*, *MIGA1, RAD51C*, *RAD51D*, and *PALB2* were more strongly associated with high-grade serous ovarian cancer whereas *MSH6, OR2T35, HELB, MYO1A* and *GABRP* were more strongly associated with the non-high-grade serous histotypes.

**Table 3:** Genes most strongly associated with epithelial ovarian cancer based on analysis of combined non-UKB and UKB data.

Case type	Gene	Minor allele freq	Odds ratio	(95% CI)	P-value	FDR	BFDP
HGSOC	*BRCA2*	0.0036	12	(8.4 - 18)	1.7 × 10^−38^	8.1 × 10^−35^	4.4 × 10^−33^
HGSOC	*BRCA1*	0.0010	40	(22 - 70)	4.8 × 10^−36^	1.2 × 10^−32^	1.5 × 10^−29^
HGSOC	*BRIP1*	0.0011	10	(5.4 - 20)	4.3 × 10^−12^	3.6 × 10^−9^	2.3 × 10^−7^
HGSOC	*RAD51C*	0.00057	12	(4.7 - 28)	8.7 × 10^−8^	5.3 × 10^−5^	0.0028
NHGSOC	*MSH6*	0.0013	11	(4.4 - 26)	1.6 × 10^−7^	8.7 × 10^−5^	0.0045
HGSOC	*RAD51D*	0.00043	13	(4.5 - 37)	2.0 × 10^−6^	9.1 × 10^−4^	0.053
NHGSOC	*OR2T35*	0.00072	15	(4.4 - 48)	1.2 × 10^−5^	0.0048	0.23
NHGSOC	*HELB*	0.00093	9.6	(3.3 - 28)	3.0 × 10^−5^	0.011	0.30
NHGSOC	*MYO1A*	0.0024	9.1	(3.1 - 27)	6.0 × 10^−5^	0.020	0.43
NHGSOC	*GABRP*	0.00072	78	(7.9 - 780)	2.0 × 10^−4^	0.060	0.92
HGSOC	*MIG1A*	0.00022	15	(3.5 - 65)	2.6 × 10^−4^	0.072	0.80
HGSOC	*PALB2*	0.0017	3.6	(1.8 - 7.4)	3.2 × 10_-_^4^	0.081	0.65
NHGSOC	*NBN*	0.0015	7	(2.4 - 21)	4.4 × 10^−4^	0.10	0.77
HGSOC	*STARD6*	0.00050	7	(2.3 - 21)	4.8 × 10^−4^	0.10	0.78
NHGSOC	*KIR3DL1*	0.00014	32	(4.5 - 230)	5.5 × 10^−4^	0.11	0.93
NMOC	*NENF*	0.00050	6.5	(2.2 - 19)	5.5 × 10^−4^	0.11	0.79
NHGSOC	*HPSE*	0.001	7.8	(2.4 - 25)	6.0 × 10^−4^	0.11	0.82
HGSOC	*OR4A47*	0.00036	8.5	(2.5 - 29)	6.5 × 10^−4^	0.11	0.84
HGSOC	*SH3BGRL*	0.00044	15	(3.2 - 73)	6.3 × 10^−4^	0.11	0.89
NHGSOC	*SHMT1*	0.0013	10	(2.6 - 42)	0.001	0.17	0.90
NHGSOC	*SALL2*	0.00022	20	(3.3 - 130)	0.0012	0.18	0.94
NHGSOC	*FASTKD5*	0.00044	33	(3.9 - 270)	0.0013	0.19	0.96
NMOC	*PLEKHG5*	0.0019	3.9	(1.7 - 9)	0.0014	0.20	0.87
HGSOC	*DQX1*	0.0017	3.5	(1.6 - 7.6)	0.0017	0.20	0.88
NHGSOC	*FAM71F1*	0.00036	16	(2.9 - 87)	0.0015	0.20	0.94
NHGSOC	*LIFR*	0.0011	11	(2.4 - 46)	0.0018	0.20	0.93
NMOC	*LIPT1*	0.00050	4.9	(1.8 - 13)	0.0017	0.20	0.89
NHGSOC	*LTBP2*	0.00050	12	(2.6 - 61)	0.0017	0.20	0.94
NHGSOC	*MMAA*	0.0011	7.5	(2.2 - 26)	0.0016	0.20	0.91
NHGSOC	*POLR2A*	0.002	5.4	(1.9 - 16)	0.0018	0.20	0.90
NHGSOC	*CD302*	0.00022	48	(4.2 - 560)	0.0019	0.20	0.97

*FDR* Benjamini-Hochberg false discovery rate, *BFDP* Bayes false discovery probability, *HGSOC* high-grade serous ovarian cancer, *NMOC* non-mucinous epithelial ovarian cancer, *NHGSOC* non-high-grade serous ovarian cancer (non-mucinous).

## Discussion

We have assembled whole exome sequencing for a large number of epithelial ovarian cancer cases and controls to investigate the role of rare, loss-of-function coding variation in the germline and risk of epithelial ovarian cancer. The exome sequencing of the non-UK Biobank cases and controls was carried out in different centres with the potential for false positive associations that are due to technical artefacts resulting in differential variant calls between cases and controls. We attempted to limit such bias by harmonising the variant calling across the different data sets with careful visual inspection of many variants using the Integrative Genomics Viewer. The lack of inflation of the test statistics for the gene-based association tests within the non-UK Biobank data suggests that any technical bias was small (if present).

**Fig. 2:**
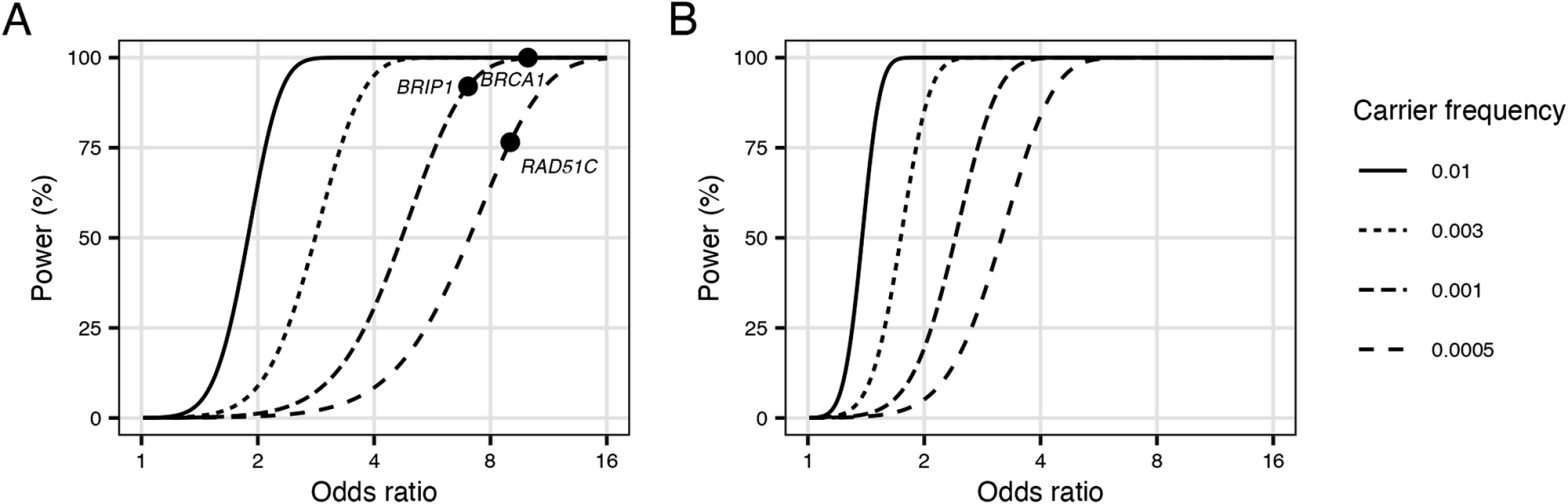
Power to detect risk alleles by carrier frequency and effect size (odds ratio) at a type 1 error probability of 5 × 10^−4^. **A** 2630 cases and 15,000 controls—the carrier frequency and effect size corresponding to BRCA1, BRIP1 and RAD51C are shown for reference. **B** 20,000 cases and 20,000 controls.

Perhaps the major limitation of this study was the limited power to detect rare variants with modest effects. Figure 2A shows the power of the available sample size to detect loss-of-function alleles by carrier frequency and effect size. Power to detect alleles with effects similar to the known genes is good, but power to detect alleles conferring odds ratios between 2 and 5 is limited. Much larger sample sizes are needed to detect more modest effects (Fig. 2B). Power may be further limited by disease heterogeneity, as histotype specific sample sizes are even smaller.

Nevertheless, we have confirmed the association of six genes known to be associated with high-grade serous ovarian cancer. There was some evidence of association of protein truncating variants in *MIGA1*, *STARD6*, *OR4A47* and *SH3BGRL* with the same histotype (FDR < 0.2). *MIGA1* encodes mitoguardin 1 which enables protein heterodimerization activity and protein homodimerization activity and is involved in mitochondrial fusion. The gene is expressed in the ovary and mitoguardin-1 and -2 promote maturation and the developmental potential of mouse oocytes by maintaining mitochondrial dynamics and functions [30]. *STARD6* encodes the StAR-related lipid transfer domain containing 6 protein which is involved in the intracellular transport of sterols and other lipids [31]. *OR4A47* encodes an olfactory receptor and *SH3BGRL* encodes SH3 domain binding glutamate-rich protein-like a scaffold protein with the potential for a variety of roles in cellular events by protein-protein interaction [32]. However, the strength of the statistical evidence for these three genes is only moderate; while the Benjamini-Hochberg False Discovery Rate was less than 0.2, the Bayes False Discovery Probability was greater than 0.5.

It is notable that of the nine genes associated with high-grade serous ovarian cancer three (*BRCA1, BRCA2, BRIP1*) were also associated with the non-high grade serous histotype (*P* < 0.05). This may be a true association, but given the limited evidence for the association of *BRCA1* and *BRCA2* with histoypes other than high-grade serous, some histotype misclassification in the data is a possible explanation. There were too few pathogenic variant carriers in the non-high-grade serous cases to estimate risk for the other six genes (*PALB2, RAD51C, RAD51D, MIGA1, SH3BGRL* and *STARD6*).

We have also confirmed the known association of the mis-match repair gene, *MSH6*, with the non-high-grade serous histotype, with another four genes associated at a False Discovery Rate of less than 0.1. *HELB* encodes DNA helicase B which catalysers the unwinding of DNA necessary for DNA replication, repair, recombination, and transcription [33]. Rare damaging variants in the gene are associated with later age at natural menopause [34]. Given the association of damaging variants with both later age at natural menopause and non-high-grade serous ovarian cancer we used Mendelian randomisation to investigate the associations of genetically determined age at natural menopause with ovarian cancer by histotypes. Genome-wide association studies have identified 290 common genetic variants associated with late age at natural menopause [35]. Published summary statistics for the association of 234 of these variants with epithelial ovarian cancer by histotype were available to use as the instrumental variable [5]. A strong association with genetically predicted late age at menopause was observed for endometrioid ovarian cancer (*P* < 0.05 for all five Mendelian randomisation methods, Supplementary Table 3), with limited evidence for clear cell ovarian cancer (*P* < 0.05 for two methods) and little evidence for the other histoypes. Power to detect an association with genetically predicted age at menopause was very good assuming a causal odds ratio per standard deviation for age at menopause of 1.2 or greater (Supplementary Fig. 1). Three of the five ovarian cases that were found to carry a loss-of-function variant in *HELB* were the endometrioid histotype, with the other two being low-grade serous. A recent WES study of 123 epithelial ovarian cancer patients identified one carrier of a loss of function variant in *HELB* [36]; this patient was diagnosed aged 25 with low-grade serous OC. Furthermore, we analysed the published data from whole-genome sequencing of tumour DNA from 59 high-grade serous, 35 clear-cell and 29 endometrioid ovarian cancers [37] for point mutations in *HELB*. Only one pathogenic variant was identified in one of the endometrioid cases. The histotype specificity of the germline and somatic association of protein-truncating variants in *HELB* together with the histotype specificity of the genetically predicted age at natural menopause association provides strong evidence that the association of protein-truncating variants in *HELB* with non-high grade serous ovarian cancer is a true positive association.

*GABRP* encodes the gamma-aminobutyric acid A receptor which is a multi-subunit chloride channel that mediates the fastest inhibitory synaptic transmission in the central nervous system. The subunit encoded by this gene is expressed in several non-neuronal tissues including the uterus and ovaries with some evidence that it is involved in cellular invasion and migration in ovarian cancer [38]. There is little evidence to link the *OR2T35* or *MYO1A* to the biology of ovarian cancer. *OR2T35* encodes olfactory receptor family 2 subfamily T member 35 and *MYO1A* encodes myosin 1A, an unconventional myosin that functions as actin-based molecular motors.

Of the other genes associated with a FDR of less than 0.2, *NBN* is perhaps the best candidate ovarian cancer susceptibility gene. It encodes nibrin, a member of the MRE11/RAD50 double-strand break repair complex involved in DNA double-strand break repair and DNA damage-induced checkpoint activation. Protein truncating variants in this gene are associated with Nijmegen breakage syndrome, an autosomal recessive condition characterised by microcephaly, growth retardation, immunodeficiency, cancer predisposition, and premature ovarian failure in females [39]. *NBN* has previously been studied using candidate-gene sequencing and no significant association was found for non-high grade serous ovarian cancer based on 444 non-high grade serous cases of which just 72 were the endometrioid histotype [1].

We have confirmed the histotype-specific associations of rare protein-truncating variants in the known epithelial ovarian cancer susceptibility genes and found a novel association for protein-truncating variants in *HELB* with risk of non-mucinous, non-high grade serous ovarian cancer. The relative risk estimate for this gene is likely to be inflated by the winner’s curse effect and may also be biased by the case ascertainment. Large case-control sequencing studies will be needed to obtain a more precise, unbiased estimate of the associated risk as well as to obtain more specific risks for the three main histotypes that comprise non-mucinous, non-high-grade serous ovarian cancer. Given our data, it is unlikely that any additional susceptibility genes exist for either epithelial ovarian cancer of all histotypes or high-grade serous ovarian cancer with the risk-allele frequency and effect-size characteristics of the known susceptibility genes. It is possible there are genes with very rare risk alleles or modest effect sizes or genes specifically associated with the less common histotypes that we have not identified. Much larger studies will be needed to identify robustly such genes.

## Supplementary information

Supplementary methods

Supplementary tablesx

## Data Availability

The accession numbers for some of the sequencing data are provided in Table 1. The results of the association statistics for the comlete set of analyses are provided in Supplementary Table 1. We are unable to post some of the raw sequencing data due to ethical and/or legal data governance constraints on the sharing of Personal Data for some of the constituent studies.
