# Supplementary material for: Exome sequencing identifies HELB as a novel susceptibility gene for non-mucinous, non-high-grade-serous epithelial ovarian cancer
Source: Eur J Hum Genet. 2025 Feb 12;33(3):297–303. doi: 10.1038/s41431-025-01786-0 (PMC11894177; doi:10.1038/s41431-025-01786-0)
Supplement: Supplementary file 1 — Supplementary methods [file 41431_2025_1786_MOESM1_ESM.pdf]

SUPPLEMENTARY MATERIAL

Supplementary Figure 1: Power to detect an association between genetically predicted age at menopause and epithelial ovarian cancer by causal effect size (odds ratio per standard deviation in age at menopause) and histotypes. Assuming 105,000 controls, 15,588 cases of high-grade serous ovarian cancer (HGSOC) , 1427 cases of clear cell ovarian cancer (CCOC) and 2,749 cases of low-grade serous ovarian cancer (LGSOC) [1]; 12% of the variation in age at menopause explained by the instrumental variable [2] and a type 1 error probability (alpha) of 0.05. The calculations are based on the method of Brion [3].

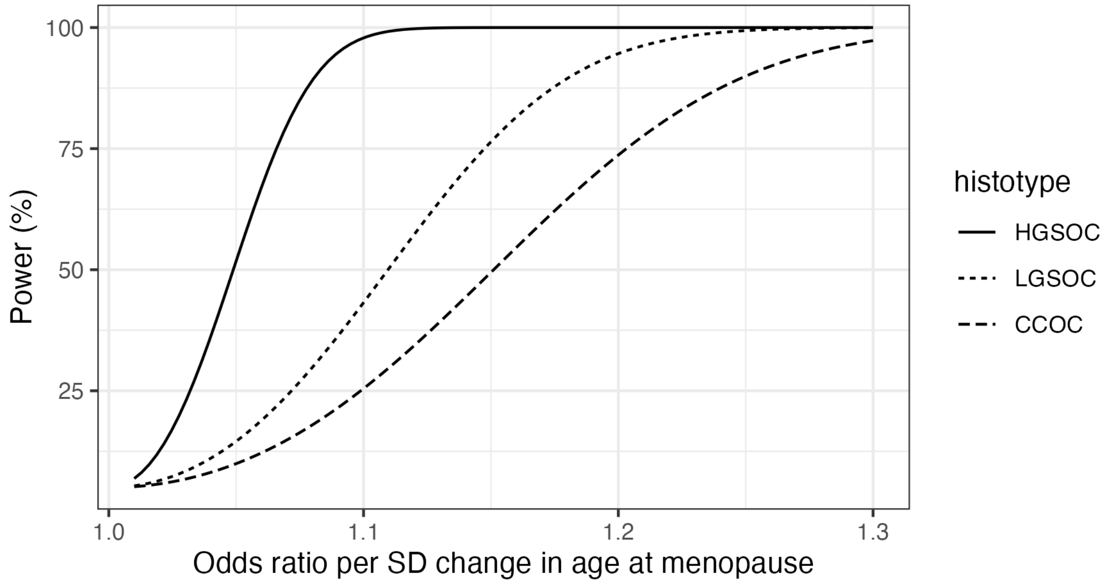

1. Dareng EO, Coetzee SG, Tyrer JP *et al*: Integrative multi-omics analyses to identify the genetic and functional mechanisms underlying ovarian cancer risk regions. *Am J Hum Genet* 2024; **111:** 1061-1083.
2. Ruth KS, Day FR, Hussain J *et al*: Genetic insights into biological mechanisms governing human ovarian ageing. *Nature* 2021; **596:** 393-397.
3. Brion MJ, Shakhbazov K, Visscher PM: Calculating statistical power in Mendelian randomization studies. *Int J Epidemiol* 2013; **42:** 1497-1501.
